# Alzheimer’s disease (AD) co-pathology in dementia with Lewy bodies (DLB): implications in the disease modification era

**DOI:** 10.1007/s00259-024-06619-8

**Published:** 2024-01-29

**Authors:** Luca Sofia, Federico Massa, Matteo Pardini, Dario Arnaldi, Matteo Bauckneht, Silvia Morbelli

**Affiliations:** 1 Department of Health Science (DISSAL), University of Genoa, Via Antonio Pastore 1, 16132 Genoa, Italy; 2 IRCCS Ospedale Policlinico San Martino, Largo Rosanna Benzi 10, 16132 Genoa, Italy; 3 Department of Neuroscience, Rehabilitation, Ophthalmology, Genetics, Maternal and Child Health (DINOGMI), Clinical Neurology, University of Genoa, Genoa, Italy

A 72-year-old man was referred to the neurologist for memory, executive, and visuospatial impairment in the last year associated with difficulty in manipulating objects, mild motor slowing, and falls. MRI highlighted temporo-lateral and posterior-parietal cortical atrophy. The syndrome was described as posterior-cortical atrophy (PCA) [1]. To differentiate between AD and DLB, the patient underwent a cardiac-[^123^I]meta-iodobenzylguanidine (MIBG) scan qualitatively normal with a borderline heart/mediastinum ratio (panel A). [^18^F]-FDG-PET showed hypometabolism in the bilateral temporo-parietal cortex and precuneus (panel B). Based on the results of MIBG and [^18^F]-FDG-PET, the patient was labeled as having PCA/AD. He was then recruited into an experimental study on atypical AD and underwent a [^18^F]-florbetaben-PET scan which confirmed a posterior pattern (early-perfusion-imaging) and was positive for brain amyloidosis (panels C, D, and E) [2]. After 1 year, the patient showed hypomimia and bradykinesia. Given the evolution of the syndrome, to further differentiate between PCA/AD and DLB presenting as PCA, a dopamine transporter imaging (DAT-SPECT) was obtained confirming dopaminergic neurodegeneration leading to a final diagnosis of DLB with comorbid AD (or mixed DLB/AD; panel F). This case emphasizes the impact of biomarkers for the etiological diagnosis of dementia. This case raises the question about the impact of sequence for the use of biomarkers on the final diagnosis: an abnormal DAT-SPECT as first examination would have led to a diagnosis of DLB and the impact of brain amyloidosis could have been neglected [3]. In clinical trials, indicators of co-pathology could be used as exclusionary criteria or to establish efficacy in a broader population in preplanned subset analyses [4–6]. 
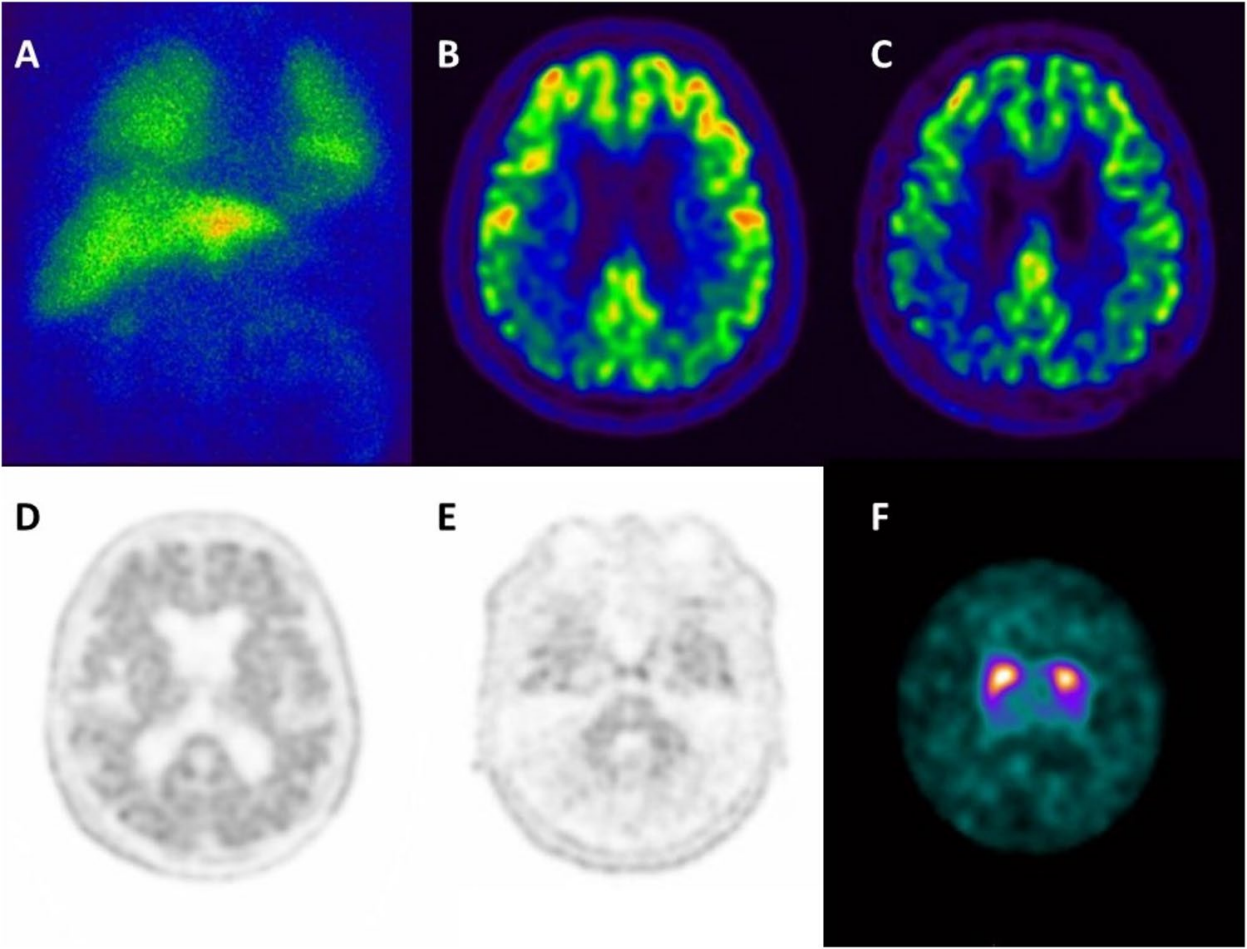


## Data Availability

Data are available upon reasonable request to the corresponding author.

## Declarations

### Open access

For related content on neuroimaging for Alzheimer’s disease, please visit: https://neuroimaging-alzheimers-disease-ime.springermedicine.com/.
